# Association between admission serum potassium and quantitative external lumbar drainage responsiveness in patients with hydrocephalus: a retrospective study

**DOI:** 10.3389/fneur.2026.1942880

**Published:** 2026-09-30

**Authors:** Yu Liu, Xiaohu Ma, Yabin Li, Qiang Fu, Tengfei Liu, Yidan Luo, Hu Qin, Yongxin Wang

**Affiliations:** 1 The Neurosurgery Department, The First Affiliated Hospital of Xinjiang Medical University, Xinjiang, China; 2 Key Laboratory of High-Incidence Disease Research in Xinjiang Region (Ministry of Education), Xinjiang, China

**Keywords:** cerebrospinal fluid dynamics, external lumbar drainage, hydrocephalus, neurological biomarkers, predictive model, serum potassium

## Abstract

**Objective:**

To evaluate the association between untreated admission serum potassium levels and quantitative responsiveness to external lumbar drainage (ELD) in patients with hydrocephalus.

**Methods:**

This single-center retrospective study included 349 unique patients with hydrocephalus after reconciliation and deduplication of clinical records collected between 2020 and 2026. Serum potassium was defined as the first measurement obtained at hospital admission before treatment. ELD responsiveness was defined as an improvement of ≥10% in Timed Up and Go or 10-m walking time, ≥3 points in the Mini-Mental State Examination (MMSE), or ≥2 points in the Montreal Cognitive Assessment (MoCA). Multivariable logistic regression was used to assess the association between potassium and ELD responsiveness after adjustment for study period and eight clinical covariates. Sensitivity analyses examined medication exposure, potassium range, and alternative outcome definitions. Model performance was evaluated using bootstrap internal validation.

**Results:**

Among 349 patients, 228 (65.3%) met the quantitative criteria for ELD responsiveness. Higher admission potassium was independently associated with greater odds of ELD responsiveness (adjusted OR per 0.1 mmol/L increase, 1.117; 95% CI, 1.038–1.201; *p* = 0.003). The association remained significant after exclusion of diuretic users (OR, 1.099; 95% CI, 1.017–1.188; *p* = 0.017). However, no significant association was observed among patients with potassium ≥3.5 mmol/L (OR, 1.043; 95% CI, 0.914–1.190; *p* = 0.536) or when a stricter definition of nonresponse was applied (OR, 1.076; 95% CI, 0.962–1.204; *p* = 0.200). The combined clinical-plus-potassium model achieved an apparent AUC of 0.757 and an optimism-corrected AUC of 0.725 (approximate 95% CI, 0.685–0.797). The corrected improvement in discrimination after adding potassium was small and uncertain.

**Conclusion:**

Higher untreated admission serum potassium was independently associated with quantitative ELD responsiveness in this heterogeneous hydrocephalus cohort. However, the association was not demonstrated within the non-hypokalemic range, and the incremental predictive contribution of potassium beyond clinical variables remained uncertain. These findings support serum potassium as a potential clinical correlate of ELD responsiveness that warrants further validation in well-characterized prospective cohorts.

## 1 Introduction

Hydrocephalus encompasses a heterogeneous group of disorders characterized by abnormal cerebrospinal fluid (CSF) dynamics. Among these, communicating hydrocephalus and idiopathic normal pressure hydrocephalus (iNPH) are commonly encountered in neurosurgical and neurological practice (1). In iNPH, the characteristic clinical presentation includes gait impairment, cognitive decline, and urinary dysfunction, frequently accompanied by radiological features such as ventriculomegaly (1). Ventriculoperitoneal shunt (VPS) is an established therapeutic intervention for appropriately selected patients (2). However, not all patients experience meaningful clinical improvement after CSF diversion, making the assessment of potentially reversible neurological dysfunction an important component of clinical decision-making.

The external lumbar drainage (ELD) test is widely used to assess short-term clinical responsiveness to CSF removal and, particularly in iNPH populations, to support decisions regarding subsequent shunt surgery. Previous studies have demonstrated the clinical utility of ELD; however, its performance can be influenced by the assessment tools, observation windows, and response criteria employed (3). Traditional gait and cognitive scales may have limited sensitivity, while a single CSF tap test can yield false-negative results (4). Prolonged ELD may improve the detection of clinically relevant functional changes, but it also increases patient burden, resource use, and procedural complexity (4). These considerations highlight the need for additional readily available clinical indicators that may complement functional assessment during ELD evaluation.

Recent research in the preoperative assessment of iNPH has increasingly incorporated objective functional measurements and biological markers. For example, digital gait analysis and digital neuropsychological assessments have shown potential for improving the evaluation of patients undergoing CSF drainage and for predicting subsequent shunt outcomes (5). CSF biomarkers have also been investigated as potential indicators of treatment responsiveness (6). However, digital functional assessments may require specialized equipment and standardized workflows, while CSF biomarker testing involves additional sample collection and laboratory procedures. Therefore, routinely available peripheral blood parameters may provide a practical complementary source of clinical information.

Serum electrolytes are routinely measured at hospital admission, and potassium (K+) plays an important role in neuronal excitability, transmembrane potential stability, and epithelial ion transport (7). Experimental studies have shown that Na+/K + -ATPase and the Na+, K+, 2Cl − cotransporter NKCC1 in the choroid plexus participate in the regulation of CSF secretion and potassium homeostasis. Manipulation of choroid plexus ion transport can alter CSF secretion and intracranial pressure, while extracellular potassium concentrations may influence the activity and transport properties of these ion-transport systems (8). These experimental findings provide a biological rationale for investigating serum potassium in relation to CSF drainage responsiveness, although peripheral serum potassium cannot be assumed to directly reflect choroid plexus transporter activity or CSF potassium homeostasis.

Based on clinical observations, we hypothesized that admission serum potassium might be associated with functional responsiveness to ELD. Serum potassium could reflect systemic physiological or metabolic conditions relevant to neurological recovery and CSF drainage response, although the underlying mechanism remains uncertain. To our knowledge, the relationship between routinely measured serum potassium and quantitatively assessed ELD responsiveness in patients with hydrocephalus has received limited investigation. In particular, whether serum potassium is independently associated with ELD response after accounting for conventional clinical, laboratory, and radiological factors remains unclear.

Therefore, the present study aimed to investigate the association between untreated admission serum potassium and quantitatively defined ELD responsiveness in a broad clinical cohort of patients with hydrocephalus. We further evaluated whether adding serum potassium to conventional pre-ELD clinical, laboratory, and radiological variables improved the classification of ELD response. Sensitivity analyses were performed to examine the robustness of the association across medication exposure, potassium range, and alternative outcome definitions. Given the retrospective design, heterogeneous study population, absence of paired CSF potassium measurements, and lack of long-term shunt outcomes, the study was intended to evaluate a clinical association and potential incremental predictive contribution rather than establish a causal mechanism or a definitive shunt-outcome prediction model.

## 2 Methods

### 2.1 Study setting and population

This single-center retrospective study was conducted at the First Affiliated Hospital of Xinjiang Medical University. Clinical records were re-extracted to complete the 2022–2026 dataset and to incorporate additional historical records from 2020 to 2022.

The original 200-patient dataset was assembled as an initial exploratory analytical sample rather than as a consecutive cohort. Because patients with a negative ELD response were less frequent among the available records, data collection for this group was continued to obtain an adequate number of nonresponders for the initial between-group analysis, while 100 responders were retained for comparison. The resulting 100/100 distribution therefore reflected the pragmatic construction of the initial analytical sample rather than the natural ELD response distribution of the source population.

The original 200-patient dataset was supplemented by 77 additional records from the later period. A separate historical extraction contained 133 records, of which 61 patients from 2022 were identified as overlapping with the later dataset. These duplicate historical records were removed, retaining a single observation for each patient. The final analytical cohort therefore comprised 349 unique patients, including 277 from the 2022–2026 dataset and 72 additional non-overlapping historical patients. The process of database reconciliation is shown in Figure 1. The 410 extracted records represent database entries before deduplication and should not be interpreted as the number of patients screened at the hospital.

**Figure 1:**
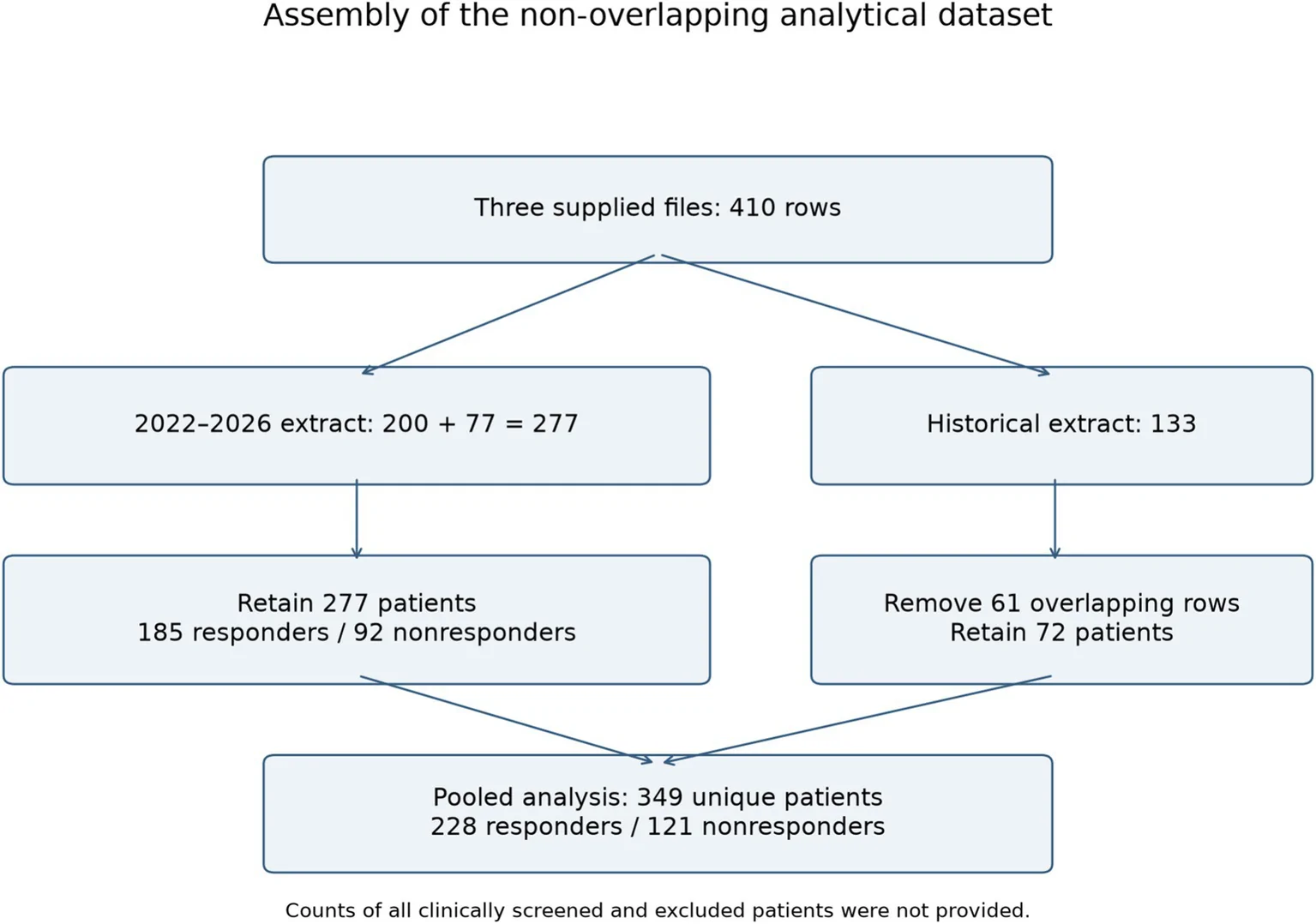
Reconciliation of extracted records. Reconciliation and deduplication of extracted clinical records. After removal of 61 overlapping records, the final analytical dataset comprised 349 unique patients. Extraction counts represent database records used to construct the analytical dataset rather than a hospital-wide screening denominator.

Eligibility required a clinical diagnosis of hydrocephalus supported by CT or MRI, completion of ELD testing, an available admission serum potassium measurement, relevant baseline clinical and imaging information, and paired gait and/or cognitive assessments. No age restriction was applied. The original eligibility framework also excluded patients with severe neurological or musculoskeletal conditions that independently prevented reliable functional assessment, inability to complete ELD testing, or an acute electrolyte disturbance requiring emergency correction before ELD. A numerical potassium threshold was not used retrospectively to define this exclusion, and no patient was excluded solely because the admission potassium concentration was <3.5 mmol/L.

A complete hospital-wide screening denominator and detailed exclusion counts could not be reliably reconstructed from the available records. Accordingly, the response proportion reported in this study reflects the analytical cohort and should not be interpreted as the overall ELD response rate of the source hospital population. The application of the emergency-electrolyte-correction criterion could not be retrospectively verified for every patient from the available dataset.

Hydrocephalus etiology and subtype were not available in a sufficiently structured form for reliable classification or subgroup analysis. Diagnostic categories were therefore not retrospectively assigned according to age or lumbar opening pressure. The study population is consequently described as a broad clinical hydrocephalus cohort rather than an iNPH-specific or adult-only population.

### 2.2 Potassium exposure and clinical measurements

The primary exposure was the first serum potassium measurement obtained after hospital admission and before treatment. Potassium values measured after supplementation or other therapeutic interventions were not substituted for the admission value. According to the clarified clinical collection procedure, patients whose potassium concentrations were within the institutional reference range did not routinely receive potassium supplementation. However, detailed patient-level treatment trajectories between the admission blood sample and ELD, including subsequent correction of low potassium, were unavailable for adjustment. The exposure therefore represents untreated admission potassium rather than the last potassium concentration immediately before ELD.

Baseline variables included sex, comorbidities, diuretic use, angiotensin-converting enzyme inhibitor or angiotensin receptor blocker (ACEI/ARB) use, Sylvian fissure enlargement, white matter hyperintensity (WMH) score, lumbar opening pressure, renal function indices, serum electrolytes, and baseline Mini-Mental State Examination (MMSE) and Montreal Cognitive Assessment (MoCA) scores. Age was available for the original 200 patients but unavailable for 149 patients from the expanded dataset and was therefore neither inferred nor imputed across collection periods. Recorded MMSE or MoCA scores of zero were retained as valid measured values.

### 2.3 ELD procedure and response assessment

ELD was performed at the L3–L4 or L4–L5 intervertebral level by an attending neurosurgeon or a more senior neurosurgeon, with lumbar opening pressure recorded at the time of catheter placement. Drainage rate and total drainage volume were individualized according to clinical circumstances, with a maximum intended drainage duration of 72 h. Drainage was terminated when clinically indicated, including the occurrence of an adverse event.

Recorded drainage duration, drainage rate, and total drained volume were summarized descriptively. These variables were not included as pretreatment predictors because they characterize the drainage intervention itself rather than baseline patient status. Because drainage rates could be adjusted or interrupted during treatment, the product of the recorded rate and duration was not assumed to equal the actual total drained volume in every patient. The available records therefore do not establish that an identical drainage protocol was applied to all patients.

Baseline functional assessments were scheduled within 24 h before catheter insertion, and post-ELD assessments were performed 24–48 h after catheter removal. Routine bedside assessments were not blinded. During research re-adjudication, the study investigators applied the predefined quantitative thresholds to the recorded pre- and post-ELD measurements. The original binary ELD label was not used to derive the revised quantitative endpoint and was retained separately only for the agreement analysis; serum potassium and imaging indices were likewise not used during re-adjudication. Counts and detailed types of ELD-related adverse events were not available for analysis.

The primary outcome was quantitative ELD responsiveness. A patient was classified as a responder if at least one of the following improvements was observed after drainage: a ≥ 10% reduction from baseline in Timed Up and Go (TUG) time, a ≥ 10% reduction in 10-m walking time, a ≥ 3-point increase in MMSE score, or a ≥ 2-point increase in MoCA score. These thresholds were used as operational study criteria and were not interpreted as universal diagnostic standards across all ages or hydrocephalus subtypes.

Patients without any observed threshold-level improvement were classified as nonresponders under the primary operational definition. Unavailable gait measurements were not assigned zero values or automatically considered treatment failures. Improvement in urinary symptoms or global neurological status derived from the original binary ELD labels was not incorporated into the primary outcome.

Because paired cognitive assessments were available for all patients, a cognitive-only outcome was examined as a sensitivity analysis. A stricter sensitivity definition of nonresponse was also applied: patients were classified as responders if any quantitative criterion was met, whereas a negative outcome required all four functional tests to be evaluable and none to reach the prespecified improvement threshold. Patients not meeting either requirement were considered unclassified in this sensitivity analysis.

### 2.4 Statistical analysis

Continuous variables were summarized as medians and interquartile ranges (IQRs), and categorical variables as counts and available-case percentages. Between-group comparisons were performed using the Mann–Whitney U test for continuous variables and Fisher’s exact test for categorical variables. Descriptive *p* values were not used for predictor selection, and missing data were reported separately.

The primary multivariable logistic regression model included 10 slope parameters: serum potassium, study period, female sex, hypertension, diuretic use, Sylvian fissure enlargement, WMH score, lumbar opening pressure scaled per 100 units, natural-log-transformed creatinine, and baseline MMSE score. Study period distinguished the 72 historical patients from the 277 patients in the later dataset. All 349 patients had complete data for variables included in the primary model.

The association between potassium and ELD responsiveness was expressed as odds ratios (ORs) per 1 mmol/L, per 0.1 mmol/L, and per one sample standard deviation, all derived from the same fitted potassium coefficient. Agreement between the original binary ELD label and the revised quantitative endpoint was evaluated using the overall agreement proportion and Cohen’s kappa, with 95% percentile confidence intervals estimated from 5,000 patient-level bootstrap samples. Neither classification was treated as an external gold standard.

The primary model specification was fixed for the current reanalysis but was not prospectively registered. No univariate significance screening or stepwise variable-selection procedure was used, and post-ELD measurements were not included as predictors. Because baseline MMSE and MoCA were strongly correlated, they were not entered simultaneously in the preferred model. Their pearson correlation and variance inflation factors (VIFs) were examined to assess multicollinearity.

Sensitivity analyses included replacing MMSE with MoCA, excluding patients receiving diuretics, restricting the analysis to patients with potassium ≥3.5 mmol/L, applying the cognitive-only and stricter outcome definitions, and additionally adjusting for ACEI/ARB use, sodium, magnesium. Age-adjusted analyses were restricted to the 200 patients with available age data. A separate analysis excluded the 15 patients known to be younger than 18 years; because age remained unavailable for 149 patients, this analysis was not interpreted as an adult-only analysis. The original binary ELD response label was examined only as an alternative outcome.

To explore the association across the potassium distribution, serum potassium was additionally categorized into tertiles based on the pooled empirical distribution, with identical values retained within the same category. The lowest tertile served as the reference group, and the same covariates as in the primary model were used for adjustment. An ordinal variable coded 1, 2, and 3 was additionally fitted to test for trend. Because of tied potassium values, tertile sample sizes were not necessarily identical. These analyses were considered supportive and were not used to define a clinical potassium threshold.

Potential nonlinearity was evaluated using a restricted cubic spline with four knots placed at the 5th, 35th, 65th, and 95th percentiles of potassium, using 3.5 mmol/L as the plotting reference. Likelihood-ratio tests assessed the overall spline association and evidence of nonlinearity. A potassium-by-study-period interaction term was also evaluated to explore potential heterogeneity between extraction periods; a nonsignificant interaction was not interpreted as evidence of equivalence.

For predictive performance, three models were evaluated: potassium alone, a clinical model containing the nine covariates other than potassium, and a combined model containing both the clinical covariates and potassium. For comparison with the previous analysis, the original 14-predictor model was also evaluated among the 200 patients with available age data. An additional pooled comparison retained the original variable structure while omitting unavailable age and adding study period; because this model included both MMSE and MoCA, it was considered a diagnostic comparison rather than the preferred specification.

Discrimination was assessed using the area under the receiver operating characteristic curve (AUC), and overall prediction error using the Brier score. Optimism correction was performed using 1,000 patient-level bootstrap resamples, with the entire fixed model-fitting procedure repeated within each resample. Approximate percentile confidence intervals for optimism-corrected performance and paired model differences were estimated using 400 outer bootstrap samples with 150 inner resamples. Apparent AUC confidence intervals were estimated using 5,000 paired patient-level bootstrap samples.

Calibration was examined using observed response rates across quintiles of predicted probability with Wilson confidence intervals, together with bootstrap-corrected logistic calibration curves. Calibration intercepts and slopes were estimated for each model. Optimism-corrected point estimates were obtained using 1,000 patient-level bootstrap resamples, and approximate 95% confidence intervals were derived using the same nested bootstrap procedure used for corrected model-performance estimates (400 outer bootstrap samples with 150 inner resamples).

Decision curve analyses were performed exploratorily across threshold probabilities from 0.10 to 0.90. Because no specific treatment decision or clinically justified threshold interval was prespecified, these analyses were not used to establish net clinical benefit or recommend a treatment threshold.

Finally, supportive continuous-outcome analyses examined post-ELD TUG, 10-m walking time, MMSE, and MoCA after adjustment for their corresponding baseline measurements and the other core covariates. Heteroscedasticity-consistent HC3 standard errors were used, with Holm correction for multiple testing across the four outcomes.

All statistical analyses were conducted in Python using NumPy, pandas, SciPy, statsmodels, scikit-learn, and Matplotlib.

## 3 Results

### 3.1 Cohort composition and outcome ascertainment

The final analytical cohort comprised 349 unique patients, of whom 228 (65.3%) met the quantitative criteria for ELD responsiveness and 121 (34.7%) did not show a threshold-level improvement. Among the responders, 18 met the gait criteria alone, 89 met the cognitive criteria alone, and 121 met criteria in both domains, corresponding to 139 gait responders and 210 cognitive responders overall (Figure 2). Paired Timed Up and Go (TUG) and 10-m walking measurements were available for 195 and 191 patients, respectively, whereas paired MMSE and MoCA assessments were available for all 349 patients.

**Figure 2:**
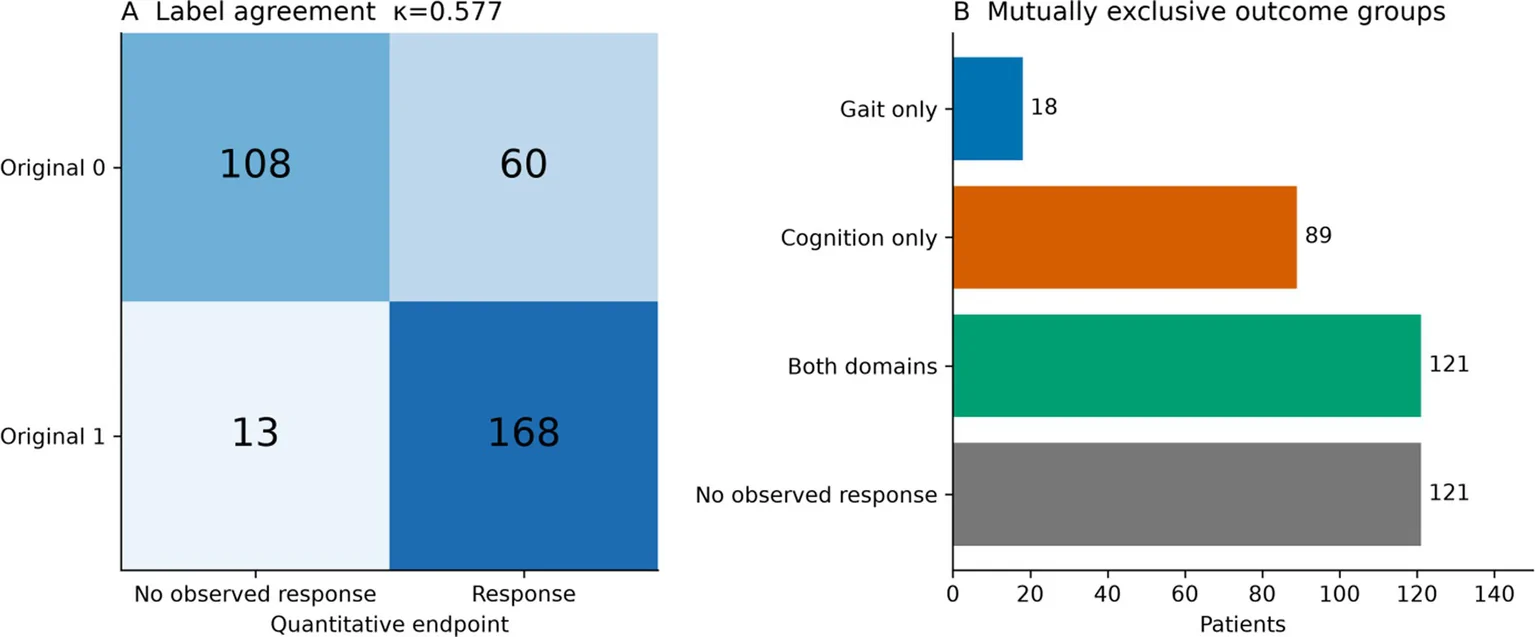
Endpoint agreement and response domains. Agreement between the original ELD classification and the quantitative endpoint and distribution of response domains. **(A)** Agreement matrix between the original binary ELD label and the revised quantitative endpoint (Cohen’s κ = 0.577). **(B)** Mutually exclusive response groups according to gait and cognitive criteria.

The cohort included 128 women (36.7%). Age was available for 200 patients, with a median of 55 years (IQR, 39.75–63.00; range, 8–81 years); 15 patients were younger than 18 years. Age was unavailable for the remaining 149 patients. Admission serum potassium was <3.5 mmol/L in 122 patients. Median potassium was higher among responders than nonresponders [3.65 mmol/L (IQR, 3.45–3.87) vs. 3.51 mmol/L (IQR, 3.33–3.76), *p* < 0.001; Table 1; Figure 3A]. Other baseline characteristics are summarized in Table 1.

**Table 1:** Baseline characteristics.

Variable	All 349	No observed response 121	Response 228	Missing	*p*
Female sex	128/349 (36.7%)	38/121 (31.4%)	90/228 (39.5%)	0	0.161
Bedridden	98/349 (28.1%)	42/121 (34.7%)	56/228 (24.6%)	0	0.047
Hypertension	155/349 (44.4%)	46/121 (38.0%)	109/228 (47.8%)	0	0.090
Diabetes mellitus	46/349 (13.2%)	17/121 (14.0%)	29/228 (12.7%)	0	0.741
Coronary disease	13/349 (3.7%)	9/121 (7.4%)	4/228 (1.8%)	0	0.014
Diuretic use	32/349 (9.2%)	13/121 (10.7%)	19/228 (8.3%)	0	0.444
ACEI or ARB use	84/349 (24.1%)	25/121 (20.7%)	59/228 (25.9%)	0	0.296
Sylvian enlargement	117/349 (33.5%)	60/121 (49.6%)	57/228 (25.0%)	0	<0.001
Age in years	55.00 [39.75, 63.00]	56.00 [40.00, 63.00]	55.00 [40.00, 63.00]	149	0.699
Recorded BMI kg/m2	23.00 [20.00, 26.95]	23.00 [20.00, 27.00]	23.00 [20.00, 26.10]	94	0.649
BUN	5.00 [3.62, 6.40]	4.73 [3.46, 6.47]	5.04 [3.81, 6.36]	0	0.340
WMH score	3.00 [1.00, 3.00]	3.00 [1.00, 3.00]	2.00 [2.00, 3.00]	0	0.864
Opening pressure	150.00 [120.00, 180.00]	130.00 [110.00, 160.00]	150.00 [120.00, 200.00]	0	<0.001
Potassium mmol/L	3.60 [3.42, 3.82]	3.51 [3.33, 3.76]	3.65 [3.45, 3.87]	0	<0.001
Sodium mmol/L	138.40 [136.00, 141.30]	138.10 [135.22, 140.00]	138.65 [136.20, 141.91]	0	0.031
Magnesium mmol/L	0.84 [0.79, 0.90]	0.83 [0.76, 0.90]	0.86 [0.80, 0.90]	0	0.040
Creatinine	57.54 [45.08, 71.04]	53.66 [39.93, 62.57]	60.17 [47.41, 73.48]	0	0.002
Baseline MMSE	11.00 [0.00, 18.00]	7.00 [0.00, 19.00]	12.00 [0.00, 18.00]	0	0.173
Baseline MoCA	10.00 [0.00, 17.00]	6.00 [0.00, 17.00]	11.00 [1.00, 17.00]	0	0.167

Values are median [Q1, Q3] or n/available (%). Age summaries describe 200 patients only. Baseline comparisons are descriptive and were not used for variable selection.

**Figure 3:**
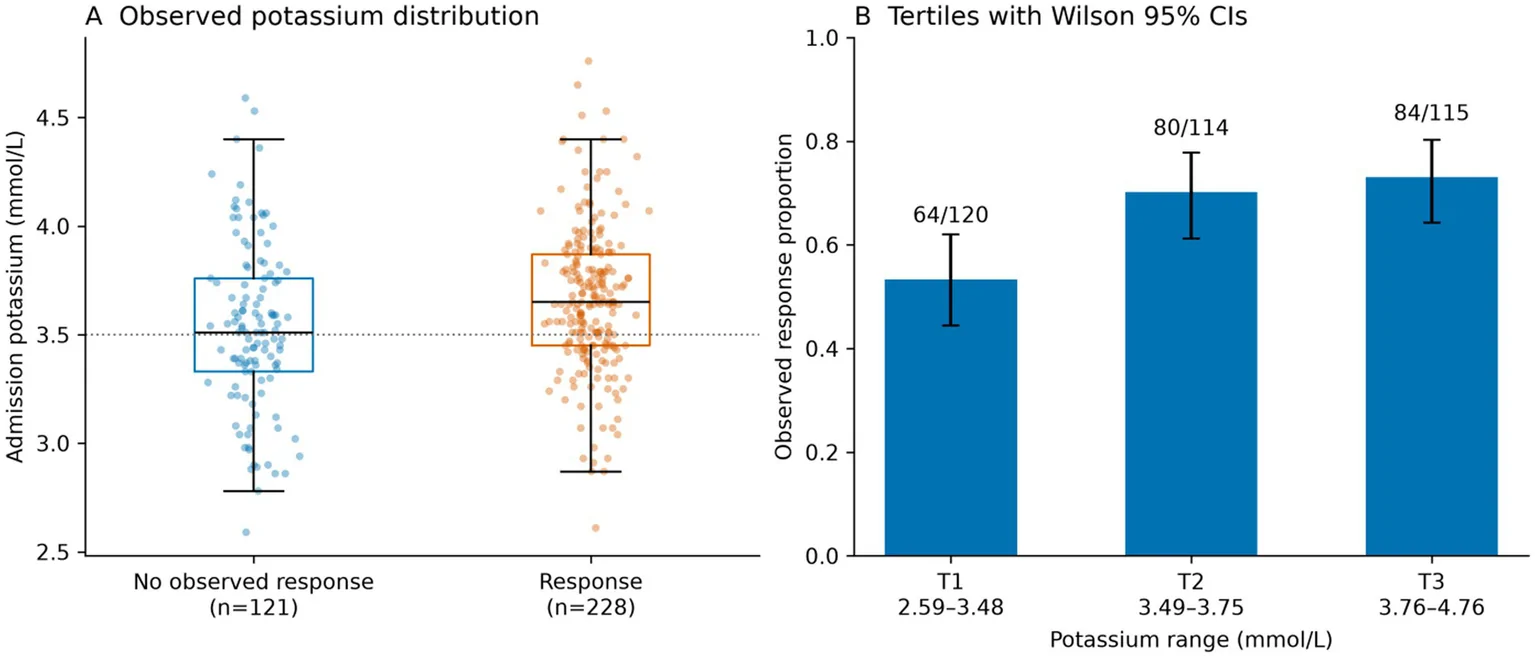
**(A)** Admission potassium distributions in responders and nonresponders. **(B)** Observed response proportions across potassium tertiles with Wilson 95% confidence intervals.

The original ELD classification and the quantitative endpoint agreed in 79.1% of patients (95% CI, 74.8–83.4%), with a Cohen’s kappa of 0.577 (95% CI, 0.493–0.658; Figure 2).

The median recorded drainage duration was 24 h (IQR, 24–48; range, 24–72 h), with a median recorded drainage rate of 6.25 mL/h (IQR, 2.20–6.25) and median total drainage volume of 150 mL (IQR, 84–300). Twenty-six duration records were corrected following source verification. After correction, 133 records showed a difference of >10% and >5 mL between the recorded total volume and the volume calculated from the recorded rate and duration, consistent with the individualized nature of drainage management.

### 3.2 Potassium association and sensitivity analyses

In the primary multivariable model, higher admission serum potassium was independently associated with greater odds of quantitative ELD responsiveness. The adjusted OR was 1.117 per 0.1 mmol/L increase in potassium (95% CI, 1.038–1.201; *p* = 0.003; Table 2; Figure 4). Expressed on alternative scales, the corresponding OR was 3.012 per 1 mmol/L increase (95% CI, 1.456–6.229) and 1.481 per one sample standard deviation of 0.356 mmol/L (95% CI, 1.143–1.919; Table 3).

**Table 2:** Complete pooled logistic model.

Predictor and scale	Beta	SE	Adjusted OR and 95% CI	*p*
Intercept	−8.60176	2.14585	Not applicable	<0.001
Female versus male	0.48131	0.27238	1.618 (0.949–2.760)	0.077
Hypertension	0.73508	0.27528	2.086 (1.216–3.577)	0.008
Diuretic use	−0.48986	0.43859	0.613 (0.259–1.447)	0.264
Sylvian enlargement	−1.15910	0.26645	0.314 (0.186–0.529)	<0.001
WMH per point	0.28664	0.15727	1.332 (0.979–1.813)	0.068
Opening pressure per 100	0.85804	0.22654	2.359 (1.513–3.677)	<0.001
Creatinine per natural-log unit	0.75325	0.38175	2.124 (1.005–4.488)	0.048
MMSE per point	0.03242	0.01716	1.033 (0.999–1.068)	0.059
Historical versus later period	−0.08982	0.30698	0.914 (0.501–1.668)	0.770
Potassium per 0.1 mmol/L	0.11026	0.03707	1.117 (1.038–1.201)	0.003

*N* = 349, responses = 228. Ten slope parameters plus intercept. For prediction, potassium must be entered as potassium/0.1 with the scaled coefficient shown. Period = 1 for historical records and 0 for later records. Binary variables are coded 1 = yes; female = 1. The raw coefficient for potassium per 1 mmol/L is 1.1025779. ORs are associations and are not risk ratios.

**Figure 4:**
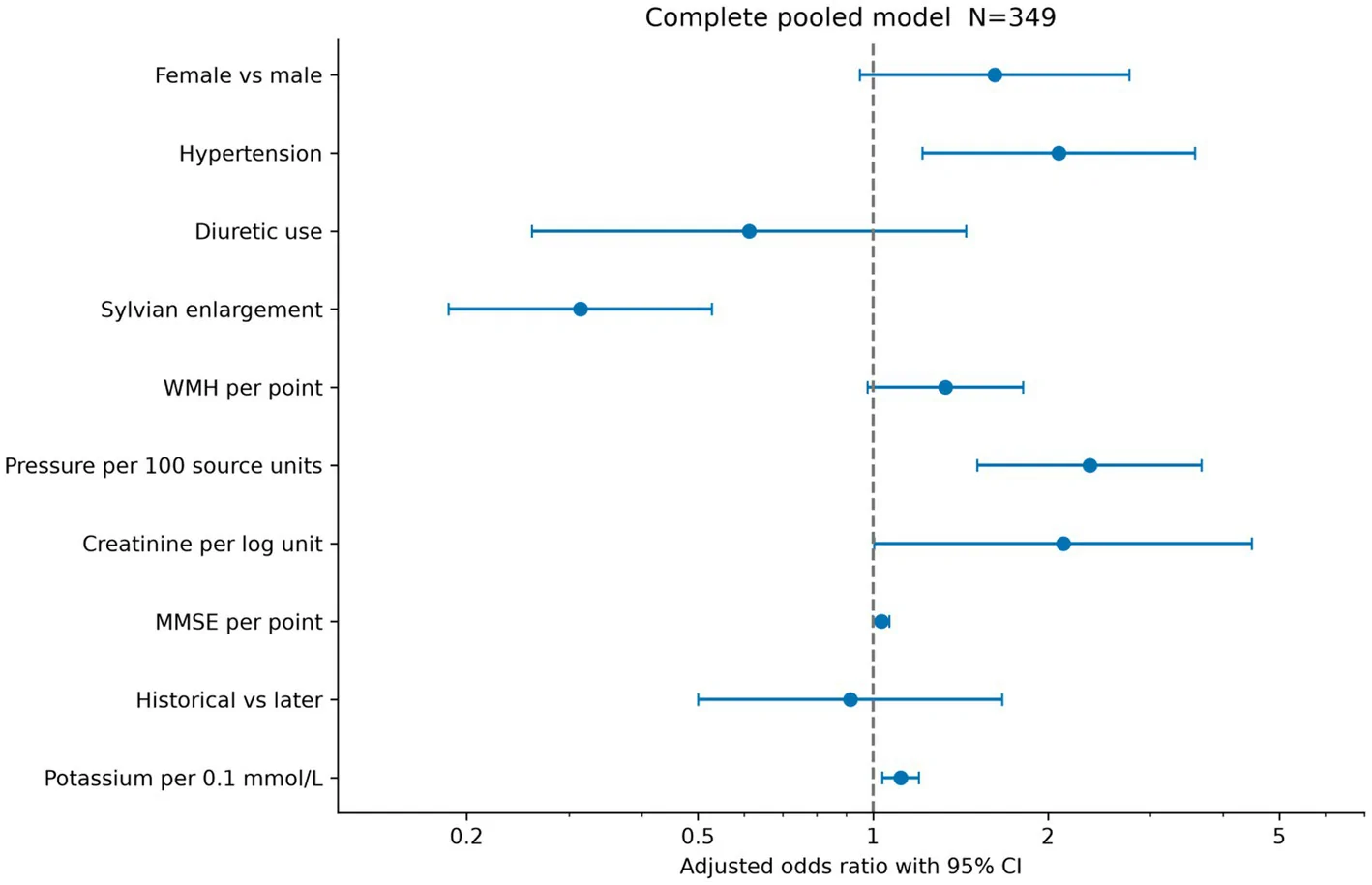
Complete pooled model. Adjusted ORs use the predictor-specific scales shown.

**Table 3:** Potassium association on three scales.

Scale	Increment mmol/L	Adjusted OR and 95% CI	*p*
Per 1 mmol/L	1.000	3.012 (1.456–6.229)	0.003
Per 0.1 mmol/L	0.100	1.117 (1.038–1.201)	0.003
Per sample SD	0.356	1.481 (1.143–1.919)	0.003

The association remained significant after excluding patients receiving diuretics (*n* = 317; OR per 0.1 mmol/L, 1.099; 95% CI, 1.017–1.188; *p* = 0.017) and when ELD response was defined using cognitive outcomes alone (OR, 1.102; 95% CI, 1.028–1.180; *p* = 0.006). In contrast, among patients with potassium ≥3.5 mmol/L, the association was not statistically significant (*n* = 227; OR, 1.043; 95% CI, 0.914–1.190; *p* = 0.536).

Under the stricter outcome definition, 228 patients were classified as responders and 41 as confirmed nonresponders, while 80 remained unclassified because all four functional tests were not evaluable. The corresponding OR was 1.076 (95% CI, 0.962–1.204; *p* = 0.200). Results were similar when MMSE was replaced by MoCA (OR, 1.118; 95% CI, 1.039–1.202; *p* = 0.003), after additional adjustment for ACEI/ARB use, sodium, and magnesium (OR, 1.111; 95% CI, 1.032–1.197; *p* = 0.005), and after exclusion of the 15 patients known to be younger than 18 years (OR, 1.123; 95% CI, 1.043–1.209; *p* = 0.002). In the 200 patients with available age data, further adjustment for age yielded an OR of 1.124 (95% CI, 1.018–1.241; *p* = 0.021; Table 4).

**Table 4:** Sensitivity analyses.

Analysis	N	Responses	OR per 0.1 and 95% CI	*p*
Primary pooled model	349	228	1.117 (1.038–1.201)	0.003
Exclude diuretic users	317	209	1.099 (1.017–1.188)	0.017
Potassium ≥3.5 mmol/L	227	162	1.043 (0.914–1.190)	0.536
Cognitive-only outcome	349	210	1.102 (1.028–1.180)	0.006
Exclude known age <18	334	218	1.123 (1.043–1.209)	0.002
Strict negative definition	269	228	1.076 (0.962–1.204)	0.200
MoCA replaces MMSE	349	228	1.118 (1.039–1.202)	0.003
Add ACEI/ARB sodium magnesium	349	228	1.111 (1.032–1.197)	0.005
Add supplement-file indicator	349	228	1.121 (1.038–1.211)	0.004
Age-adjusted analysis among patients with available age data	200	115	1.124 (1.018–1.241)	0.021

Strict negatives require all four tests; 80 patients remain unclassified. Excluding known minors leaves 149 patients with unknown age. No claim of equivalence or universal robustness follows from these analyses.

Observed response proportions increased across potassium tertiles (Figure 3B), and this pattern remained evident after multivariable adjustment. The lowest tertile (2.59–3.48 mmol/L) included 64 responders among 120 patients, compared with 80 of 114 in the middle tertile (3.49–3.75 mmol/L) and 84 of 115 in the highest tertile (3.76–4.76 mmol/L). Relative to the lowest tertile, the adjusted OR was 1.893 (95% CI, 1.052–3.404; *p* = 0.033) for the middle tertile and 2.299 (95% CI, 1.258–4.201; *p* = 0.007) for the highest tertile. The adjusted OR per increasing tertile was 1.533 (95% CI, 1.132–2.076; *p* = 0.006; Table 5; Figure 5).

**Table 5:** Potassium tertiles and trend.

Group and range mmol/L	Responses / N	Adjusted OR and 95% CI	*p*
T1 2.59–3.48	64/120	Reference	—
T2 3.49–3.75	80/114	1.893 (1.052–3.404)	0.033
T3 3.76–4.76	84/115	2.299 (1.258–4.201)	0.007

Trend per ordered tertile: OR 1.533 (95% CI 1.132–2.076), *p* = 0.006. Ties remain in the same tertile; group sizes therefore differ. Adjustment uses the same core covariates excluding continuous potassium.

**Figure 5:**
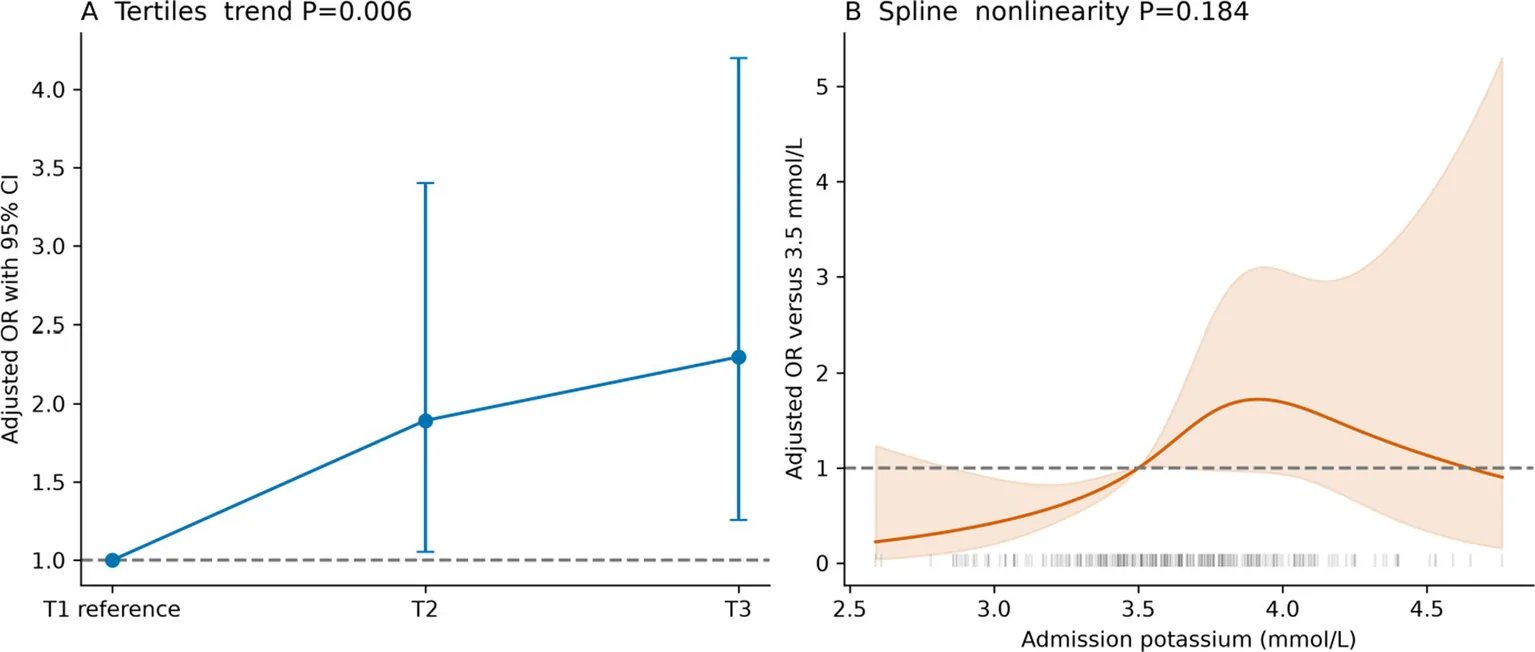
Potassium tertiles and spline. Association between admission serum potassium and quantitative ELD responsiveness. **(A)** Adjusted odds ratios across potassium tertiles, with the lowest tertile as reference (P for trend = 0.006). **(B)** Restricted cubic spline analysis referenced to 3.5 mmol/L; overall *p* = 0.005 and P for nonlinearity = 0.184. Shaded areas indicate 95% confidence intervals.

Restricted cubic spline analysis used knots at potassium concentrations of 2.980, 3.498, 3.740, and 4.232 mmol/L. The overall potassium association was significant (*p* = 0.005), whereas the test for nonlinearity was not (*p* = 0.184; Figure 5). The potassium-by-study-period interaction was also not significant (*p* = 0.578).

### 3.3 Model specification and performance

Baseline MMSE and MoCA scores were strongly correlated (Pearson r = 0.966). The primary model therefore included MMSE without simultaneous inclusion of MoCA. Variance inflation factors for variables in the primary model ranged from 1.039 to 1.478, indicating limited multicollinearity. Full regression coefficients are presented in Table 2.

Potassium alone yielded an apparent AUC of 0.611 (95% CI, 0.544–0.675) and an optimism-corrected AUC of 0.612 (approximate 95% CI, 0.531–0.674). The clinical model had an apparent AUC of 0.734 (95% CI, 0.679–0.788) and a corrected AUC of 0.704 (approximate 95% CI, 0.664–0.774). After potassium was added, the combined model achieved an apparent AUC of 0.757 (95% CI, 0.705–0.808) and a corrected AUC of 0.725 (approximate 95% CI, 0.685–0.797). The corresponding corrected Brier scores were 0.203 for the clinical model and 0.199 for the combined model (Table 6; Figure 6).

**Table 6:** Pooled model performance.

Model	Apparent AUC and 95% CI	Corrected AUC and approximate 95% CI	Corrected Brier and approximate 95% CI	Corrected calibration intercept (95% CI)	Corrected calibration slope (95% CI)
Potassium alone	0.611 (0.544–0.675)	0.612 (0.531–0.674)	0.221 (0.200–0.236)	−0.002 (−0.021 to 0.014)	1.135 (−2.162 to 2.042)
Clinical	0.734 (0.679–0.788)	0.704 (0.664–0.774)	0.203 (0.179–0.218)	−0.003 (−0.023 to 0.018)	0.837 (0.791 to 0.885)
Clinical plus potassium	0.757 (0.705–0.808)	0.725 (0.685–0.797)	0.199 (0.172–0.214)	−0.002 (−0.026 to 0.017)	0.834 (0.791 to 0.878)

Apparent intervals use 5,000 patient bootstrap samples of fixed fitted predictions; they do not constitute validation. Corrected intervals use nested refitting (400 outer ×150 inner). Primary correction uses 1,000 samples. Corrected ΔAUC = 0.02165 (−0.00001 to 0.05265); ΔBrier = −0.00462 (−0.01463 to 0.00065). Calibration parameter confidence intervals were estimated using 400 outer bootstrap samples with 150 inner resamples; failed inner fits were omitted without artificial replacement.

**Figure 6:**
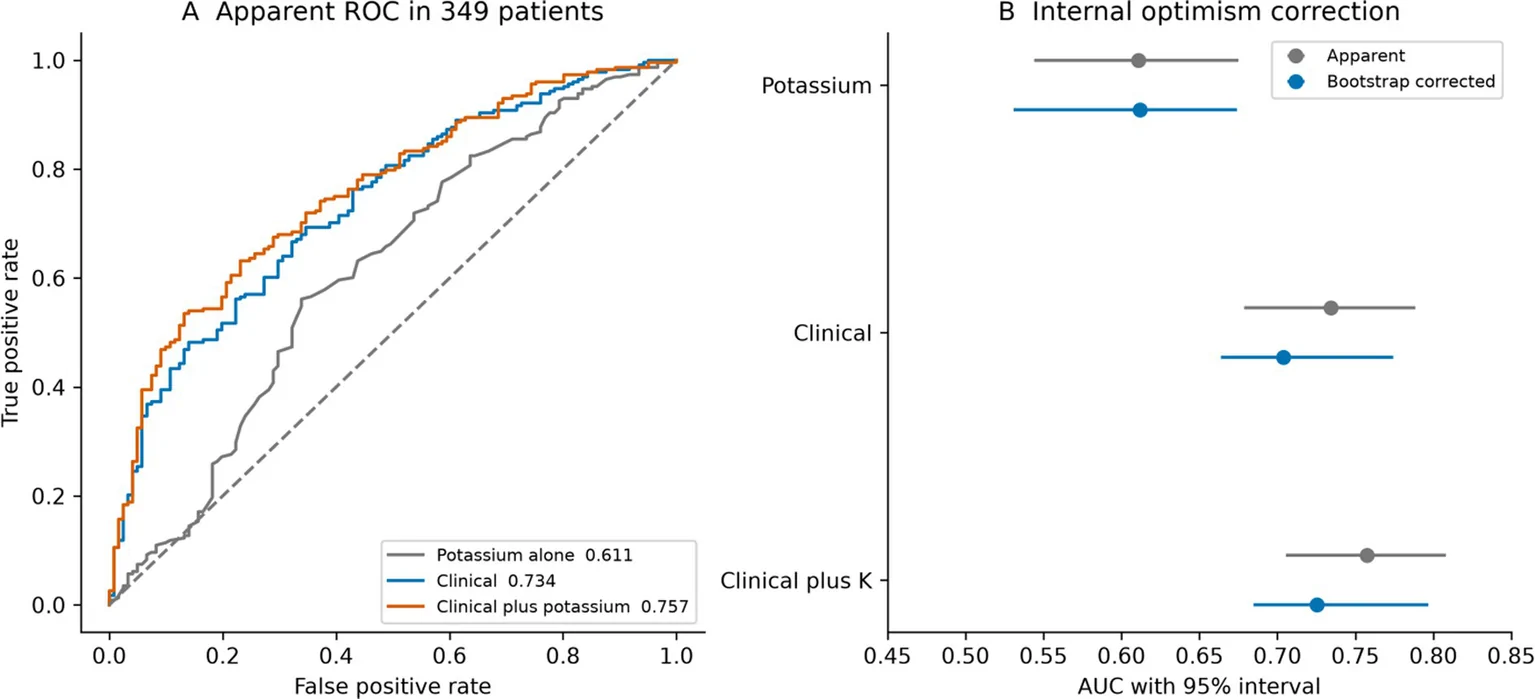
**(A)** Apparent ROC curves for the potassium-only, clinical, and clinical-plus-potassium models. **(B)** Apparent and bootstrap-corrected AUC estimates with 95% confidence intervals for the three models.

Adding potassium significantly improved overall model fit (likelihood-ratio χ^2^ = 9.298, *p* = 0.002). The optimism-corrected increase in AUC was 0.02165 (approximate 95% CI, −0.00001 to 0.05265), while the change in Brier score was −0.00462 (95% CI, −0.01463 to 0.00065; Table 7). Thus, the combined model showed a modest improvement in discrimination and overall prediction error, although the confidence intervals for the incremental performance measures included the null value.

**Table 7:** Increment from adding potassium.

Measure	Estimate	95% interval or *p*
Likelihood ratio chi-square	9.298	*p* = 0.002
Corrected AUC difference	0.02165	−0.00001 to 0.05265
Corrected Brier difference	−0.00462	−0.01463 to 0.00065

Likelihood-ratio significance measures fit. Corrected increments do not establish a clinically useful gain.

The apparent ROC curves and bootstrap-corrected discrimination estimates for the three models are shown in Figure 6. Calibration results are shown in Figure 7. The bootstrap-corrected calibration intercepts were −0.002 (95% CI, −0.021 to 0.014), −0.003 (95% CI, −0.023 to 0.018), and −0.002 (95% CI, −0.026 to 0.017) for the potassium-only, clinical, and combined models, respectively. The corresponding bootstrap-corrected calibration slopes were 1.135 (95% CI, −2.162 to 2.042), 0.837 (95% CI, 0.791 to 0.885), and 0.834 (95% CI, 0.791 to 0.878), respectively. The wide confidence interval for the potassium-only calibration slope indicates substantial uncertainty in its calibration, whereas the clinical and combined models showed more stable calibration estimates. Exploratory decision curves for the three models are shown in Figure 8.

**Figure 7:**
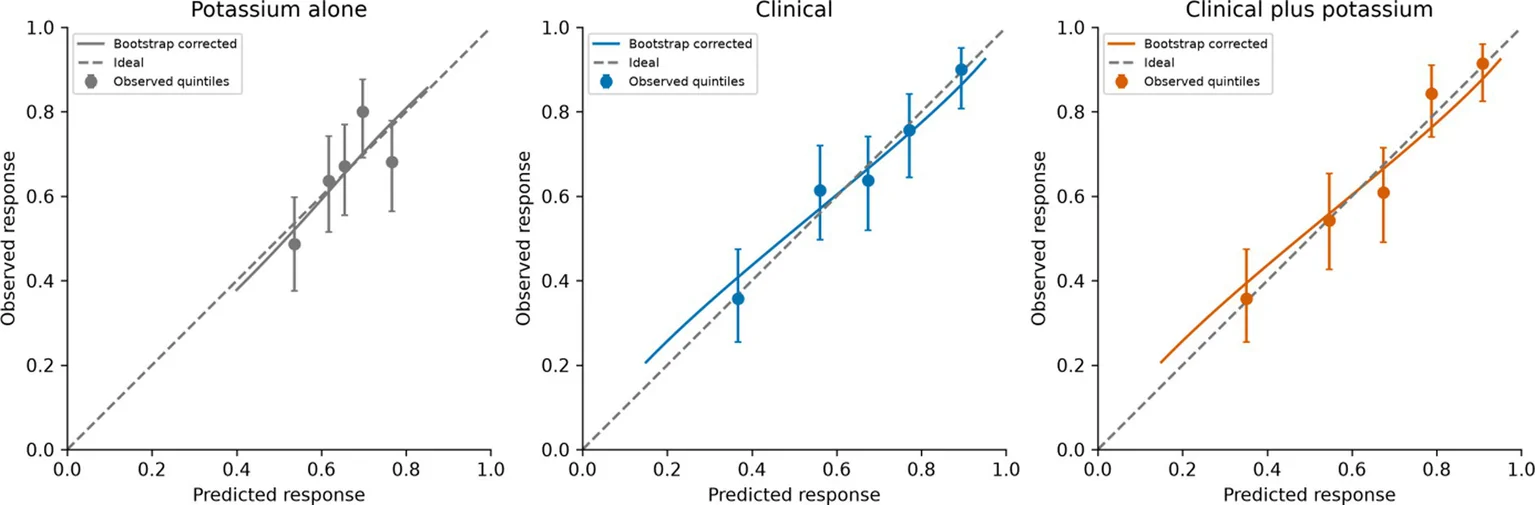
Three model calibration. Observed quintiles with Wilson intervals and bootstrap-corrected logistic calibration. In-sample calibration is not external validation.

**Figure 8:**
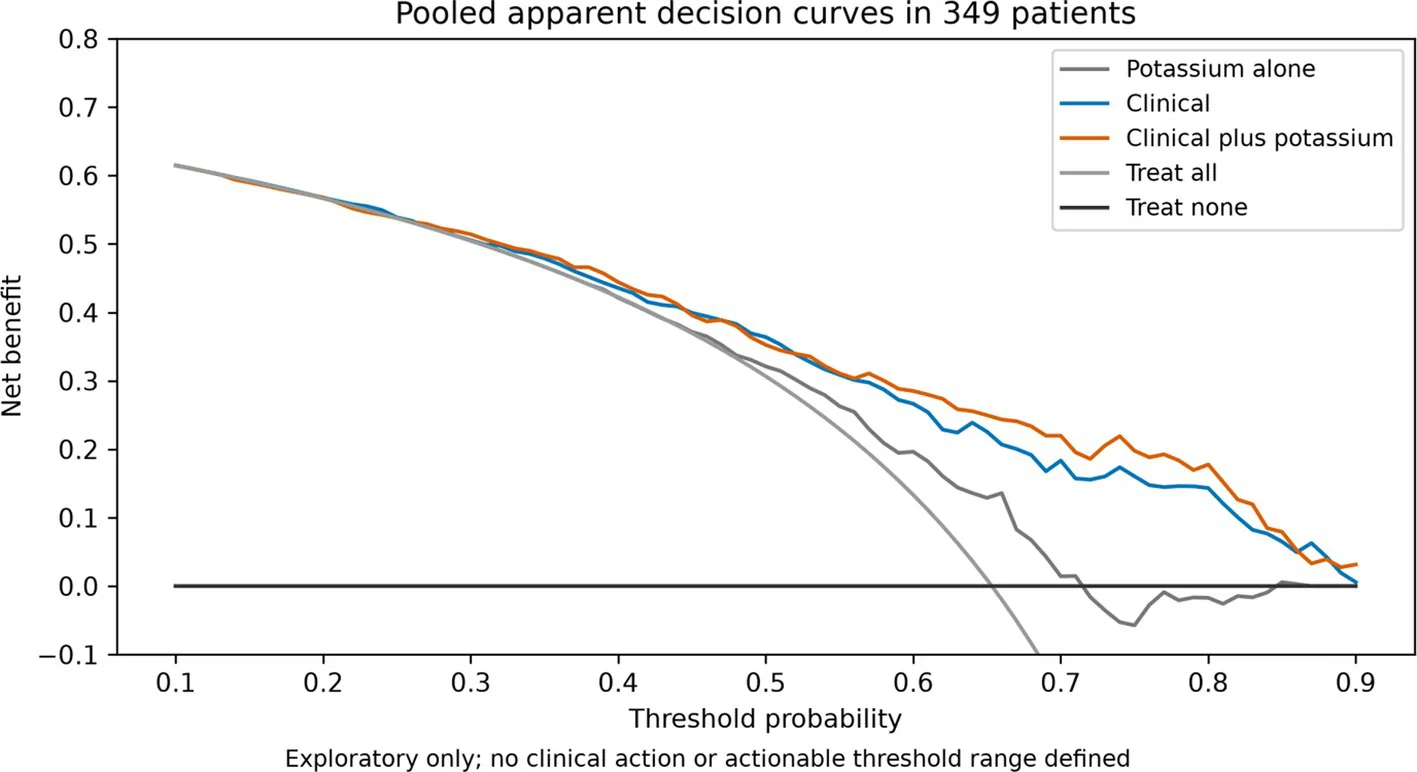
Exploratory decision curves. Pooled apparent curves only. No clinical decision threshold was established.

For comparison with the previous analysis, the original 14-predictor model applied to the 200 patients with complete age information yielded an adjusted OR of 1.132 per 0.1 mmol/L higher potassium (95% CI, 1.023–1.254; *p* = 0.017). When the original model structure was applied to the full cohort after omitting age and adding study period, the corresponding OR was 1.114 (95% CI, 1.035–1.200; *p* = 0.004).

In continuous-outcome analyses, each 0.1 mmol/L increase in admission potassium was associated with an adjusted post-ELD difference of −0.876 s in TUG time (95% CI, −1.400 to −0.353), −2.535 s in 10-m walking time (95% CI, −4.030 to −1.041), +0.197 points in MMSE (95% CI, 0.051–0.343), and +0.217 points in MoCA (95% CI, 0.052–0.381). Holm-adjusted *p* values were 0.004, 0.004, 0.017, and 0.017, respectively. These continuous-outcome findings were directionally consistent with the primary binary-response analysis.

## 4 Discussion

This study investigated the association between admission serum potassium levels and quantitatively defined ELD responsiveness in patients with hydrocephalus. In the expanded and deduplicated cohort of 349 unique patients, 228 (65.3%) met the quantitative response criteria and 121 showed no threshold-level improvement. Serum potassium levels were higher in responders than in nonresponders, with median concentrations of 3.65 mmol/L (IQR, 3.45–3.87) and 3.51 mmol/L (IQR, 3.33–3.76), respectively (*p* < 0.001). After adjustment for study period and clinically relevant pre-ELD variables, higher admission potassium remained independently associated with ELD responsiveness, with an adjusted OR of 1.117 per 0.1 mmol/L increase (95% CI, 1.038–1.201; *p* = 0.003). The association remained significant after excluding diuretic users and when response was defined using cognitive outcomes alone. However, no significant association was observed among patients with potassium ≥3.5 mmol/L. Serum potassium alone also showed limited discrimination, with an apparent AUC of 0.611 and an optimism-corrected AUC of 0.612. These findings suggest that serum potassium is better considered a complementary clinical correlate than a standalone predictor of ELD responsiveness.

Previous research has indicated that although the ELD test can support clinical assessment and selection of patients for ventriculoperitoneal shunt (VPS), predictors of short-term responsiveness to CSF drainage do not necessarily correspond directly to predictors of long-term post-shunt benefit (9). This discrepancy may reflect differences in drainage rate and volume, timing of functional assessment, natural symptom fluctuations, comorbidities, and delayed improvement in cognitive or urinary symptoms (10). ELD is generally considered a useful component of the clinical evaluation pathway, but short-term responsiveness and postoperative outcome represent related yet distinct clinical endpoints (11). Accordingly, the present study focused specifically on quantitative short-term ELD responsiveness rather than postoperative shunt outcome.

Compared with previous studies, a distinctive aspect of the present work is its focus on a routinely available peripheral blood parameter rather than exclusively on functional testing or CSF biomarkers. Current research on ELD and CSF drainage has largely emphasized optimization of gait and cognitive assessment. For example, Cai et al. reported that digital neuropsychological and gait assessments showed strong performance in predicting subsequent shunt outcomes in patients with iNPH (5). Other studies have similarly emphasized the clinical value of standardized post-drainage functional changes for patient selection (12). In contrast, serum potassium alone showed only modest discriminative ability in our cohort. The apparent AUC increased from 0.734 for the clinical model to 0.757 after potassium was added, while the optimism-corrected AUC increased from 0.704 to 0.725. Model fit also improved significantly (likelihood-ratio χ^2^ = 9.298, *p* = 0.002). However, the optimism-corrected ΔAUC was 0.02165 (approximate 95% CI, −0.00001 to 0.05265), indicating that the incremental gain in discrimination was modest and remained uncertain. These findings support the view that serum potassium may provide additional information alongside conventional clinical and radiological variables, but is unlikely to replace functional assessment, neuroimaging, or CSF dynamic evaluation.

The mechanistic interpretation of the observed association remains hypothesis-generating. CSF formation is now understood as an active secretory process involving multiple ion transport systems in the choroid plexus epithelium rather than a purely passive osmotic phenomenon (13). NKCC1 (Na+, K+, 2Cl − cotransporter 1), located on the apical membrane of choroid plexus epithelial cells, contributes to CSF secretion and potassium homeostasis (14). Experimental manipulation of choroid plexus NKCC1 can alter CSF secretion and intracranial pressure, supporting an important role for ion transport in CSF homeostasis. Na+/K + -ATPase is also highly expressed on the apical membrane of choroid plexus epithelial cells and contributes to the ionic gradients required for CSF secretion (8). Together, these transport systems provide a plausible biological context linking potassium regulation with CSF physiology (8).

Previous experimental studies have further suggested that chronic alterations in systemic potassium balance can influence potassium transport by the choroid plexus. In hypokalemic animal models, reduced ouabain-sensitive potassium uptake has been observed in choroid plexus tissue, suggesting adaptation of epithelial potassium transport under sustained potassium depletion (15). Changes in extracellular or CSF potassium concentrations may also modify Na+/K + -ATPase activity and NKCC1-mediated ion transport, thereby contributing to maintenance of CSF potassium homeostasis (15, 16). These observations provide biological support for investigating potassium status in disorders involving altered CSF dynamics.

One possible interpretation is that admission serum potassium may reflect, at least in part, the broader metabolic and ion-regulatory state of patients undergoing CSF drainage. Differences in potassium homeostasis could potentially coexist with differences in choroid plexus ion transport, systemic illness, medication exposure, or physiological reserve, any of which might influence short-term functional recovery following ELD (17, 18). This possibility is particularly relevant because the association remained detectable after excluding diuretic users. At the same time, the absence of a significant association among patients with potassium ≥3.5 mmol/L suggests that the relationship may be more pronounced at lower potassium concentrations rather than representing a uniform effect across the entire physiological range. The tertile and spline analyses were consistent with an overall potassium-response association but did not identify a clear clinical threshold.

The present findings should therefore not be interpreted as evidence that serum potassium directly measures choroid plexus transport capacity. This study did not include paired CSF potassium measurements, direct assessment of NKCC1 or Na+/K + -ATPase activity, or experimental manipulation of potassium status. It therefore remains unknown whether lower serum potassium contributes directly to reduced ELD responsiveness or simply accompanies other physiological factors associated with clinical recovery. Similarly, the present results do not establish that potassium supplementation would improve the response to CSF drainage. Future studies combining serum and CSF electrolyte measurements with CSF dynamic parameters and molecular assessment of choroid plexus ion transport may help clarify the biological basis of the observed association.

Several features of the present study should be considered when interpreting its clinical implications. The cohort represents a broad clinical hydrocephalus population rather than an exclusively iNPH population, and hydrocephalus etiological subtype was not available for reliable stratified analysis. Age was also unavailable for part of the expanded cohort. In addition, the study evaluated short-term ELD responsiveness rather than subsequent shunt outcome, and patient-level treatment trajectories between the admission potassium measurement and ELD were not fully available. Across sensitivity analyses, the association remained significant after exclusion of diuretic users, with cognitive-only outcome assessment, alternative cognitive adjustment, and age adjustment among patients with available age data; however, it was not demonstrated in the non-hypokalemic subgroup or under the stricter outcome definition.

In conclusion, higher untreated admission serum potassium was independently associated with quantitatively defined ELD responsiveness in this broad clinical cohort of patients with hydrocephalus. Serum potassium alone had limited discriminative ability, while its addition to conventional clinical variables produced a modest improvement in model performance. The association was not demonstrated within the non-hypokalemic subgroup, suggesting that its clinical relevance may be greater among patients with lower potassium concentrations. Serum potassium may therefore represent an inexpensive and readily available supplementary marker rather than an independent decision-making tool. Prospective multicenter studies incorporating etiological classification, standardized ELD assessment, serum and CSF electrolytes, CSF dynamic measurements, and postoperative shunt outcomes are warranted to determine its clinical utility and biological relevance.

## Data Availability

The original contributions presented in the study are included in the article/supplementary material, further inquiries can be directed to the corresponding author.
